# A case of adnexal cutaneous leishmaniasis in Washington DC

**DOI:** 10.1186/s12348-024-00423-z

**Published:** 2024-08-23

**Authors:** Sinan Akosman, Heeyah Song, Paul Sheils, Tamer Mansour, Keith J. Wroblewski, Lamise Rajjoub

**Affiliations:** Department of Ophthalmology, George Washington University School of Medicine, 2150 Pennsylvania Avenue Suite 2A, Washington, DC USA

**Keywords:** Cutaneous leishmaniasis, Parasites, Punch biopsy, Non-endemic, Ocular leishmaniasis, Washington DC, United States

## Abstract

**Purpose:**

To report a rare non-endemic case of *Leishmania aethiopica* in Washington DC.

**Case report:**

A 68-year-old female presented for a routine examination with a complaint of right upper eyelid lesions for the past 5 months. On examination, a cluster of elevated and erythematous lesions extending from the medial canthus to the brow area of the right eye were seen. Initial treatment with Valtrex based on a suspected viral etiology failed. Although a biopsy was recommended at this time, the patient declined, and subsequent workup included nasolacrimal duct irrigation, blood work to rule out autoimmune etiology, a course of doxycycline, and an MRI, which yielded no improvement. Upon progression of the lesions into persistent plaques on the eyelids, a punch biopsy was performed, confirming leishmaniasis. The patient was then started on a 28-day course of oral miltefosine which led to complete resolution of her symptoms.

**Conclusion:**

This case underlines the importance of a broad differential including non-endemic diseases, particularly in urban areas with frequent patient travel. Furthermore, the delayed punch biopsy in this case highlights the importance of patient counseling to ensure prompt diagnosis and treatment.

## Introduction

Leishmaniasis is an infectious disease caused by the protozoan parasites of the *Leishmania* genus. *Leishmania* are transmitted by the bite of infected female phlebotomine sandflies, and known reservoirs of this parasite can be humans as well as animals [1]. The World Health Organization (WHO) estimates between 700,000 to 1,000,000 new cases of leishmaniasis worldwide annually, with several endemic regions in the Americas, Mediterranean, Europe, and South-East Asia [2].

The clinical manifestations of leishmaniasis are largely categorized into cutaneous leishmaniasis (CL), mucocutaneous leishmaniasis, or visceral leishmaniasis. CL is the most common form of leishmaniasis and typically presents with skin lesions or ulcers on extremities. Facial lesions are typical in CL, yet eyelid and periorbital involvement is relatively rare, accounting for only about 2% of all CL cases [1, 3, 4].

Diagnosing adnexal leishmaniasis poses a challenge due to its similarity to more common lesions, such as preseptal cellulitis, orbital pseudotumor, sarcoidosis, ophthalmic zoster, allergic reactions, edema due to hypoproteinemia, and thyroid-associated ophthalmopathy [4–6]. In this report, we describe the clinical features, diagnosis, and treatment of a patient with CL presenting with right upper eyelid lesions.

## Case report

**Fig. 1:**
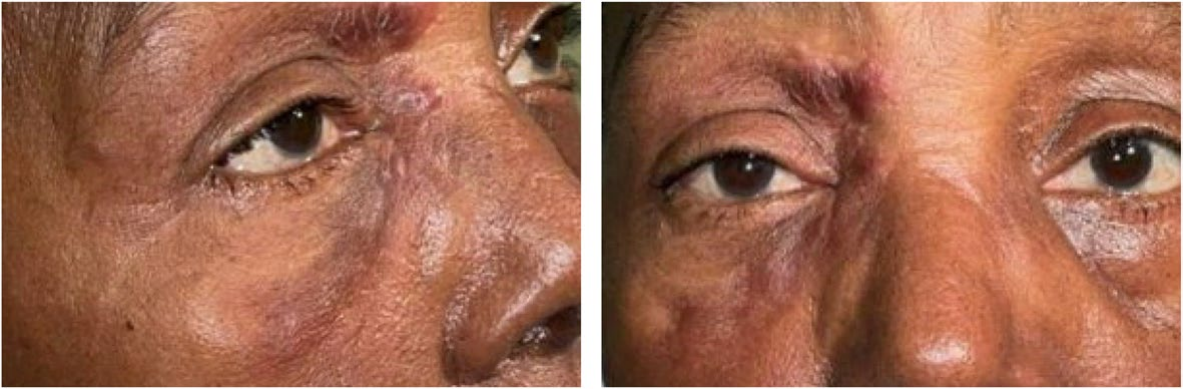
Shows an external photograph of her right eye which demonstrates erythematous, infiltrative plaques that extend across the medial canthal region up toward the brow

A 68-year-old female presented for a routine examination with a complaint of right upper eyelid lesions for the past 5 months. The patient was of Ethiopian origin and reported recent travel to Ethiopia within the last year. On examination, a cluster of elevated lesions measuring 7 mm × 13 mm near the medial canthus of the right eye were seen extending up to the brow area (Fig. 1). The lesions were erythematous with associated soft tissue edema and non-tender to the touch. The lesions were only limited to the right eye. Suspecting a viral or shingles-related cause, a two-week course of Valtrex was prescribed, but it yielded no improvement. In a subsequent evaluation two months later, the lesions worsened with increased erythema, size, and edema, infiltrating deeper in the periocular area. Although biopsy was offered multiple times, the patient repeatedly declined despite multiple counseling attempts. Subsequently the patient was then referred to oculoplastic clinic for further evaluation. Nasolacrimal duct was irrigated to rule out any lacrimal involvement which was negative. Patient was subsequently started on a course of doxycycline and extensive blood work including angiotensin converting enzyme (ACE), immunoglobulin G4 (IgG4), C1 esterase, rheumatoid factor (RF) and antineutrophil cytoplasmic antibodies (ANCA) was done to rule out any autoimmune etiology without much yield. Concerned for possible neoplastic etiology, the MRI face/orbit was completed, which showed soft tissue swelling in the right preseptal and pre-maxillary region extending across the nasion with enhancement of levator palpebrae muscle, right lacrimal gland and nasolacrimal duct. The MRI findings were highly suggestive of inflammatory or infectious etiology. Unfortunately, the lesions persisted. Differentials considered at this juncture included sarcoid/orbital and pseudotumor, with cellulitis being less likely.

The patient was then referred to dermatology. During the evaluation, persistent plaques on the right eyelids now involving the bridge of the nose were seen. At this point, patient agreed to a punch biopsy, which showed intracytoplasmic infection most consistent with leishmaniasis. Subsequently, the patient was referred to the National Institutes of Health (NIH). A second biopsy of the right periorbital lesion was done at NIH which was PCR positive for *Leishmenia aethiopica*. Patient was offered IV amphotericin and a 28-day course of oral miltefosine; our patient elected to proceed with miltefosine, resulting in complete resolution of her lesions without any reported adverse events.

### Pathology

**Fig. 2:**
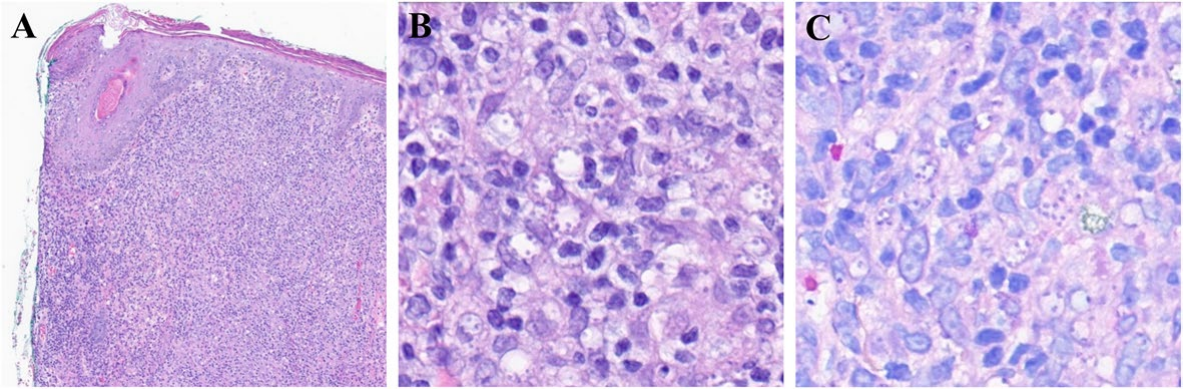
Histopathologic features consistent with intracellular infection. **A** Dense pan-dermal infiltrate (hematoxylin–eosin staining, original magnification × 4); **B** Parasitized histiocytes with staining of Leishmania amastigotes (PAS, original magnification × 40); and **C** Parasitized histiocytes with staining of Leishmania amastigotes (Giemsa, original magnification × 40)

The skin biopsy from the right root of the nose demonstrated a dense pan-dermal infiltrate composed of primarily lymphocytes and histiocytes with scattered other inflammatory cells on low magnification (Fig. 2, panel A).

On PAS (Fig. 2, panel B) and Giemsa (Fig. 2, panel C) special staining, within the lymphocytes, vacuoles are identified with small organisms arranged around the periphery of the vacuoles. No gelatinous capsule is identified. These organisms were not visualized on GMS special staining.

Taken together, the findings are consistent with an intracellular infection, such as leishmaniasis or histoplasmosis, among others, in the correct clinical context. Correlation with serologic study and deep tissue culture, if indicated, is recommended. The case was discussed with dermatopathology, who agreed with the diagnosis as above.

## Discussion

This case highlights the growing challenge and significance of considering leishmaniasis in physicians' diagnostic considerations in non-endemic regions, including the United States. In the recent years, CL has a rising global prevalence due to increasing migration from endemic areas, which requires healthcare professionals around the world to be able to effectively recognize leishmaniasis and its complications [7].

In our case, the unusual location of the lesions led to a delay in diagnosis and management. Periorbital lesions in cutaneous leishmaniasis are thought to be rare due to the movement of the eyelids which deters the bite of the fly vector [8, 9]. In a study of over 1,700 CL lesions of patients in Turkey, only 1.93% of lesions were located on the eyelid and the periorbital region [3]. Additionally, adnexal Leishmaniasis commonly presents as a chalazion-like lesion which can often bear resemblance to a multitude of other cutaneous lesions such as those from recurrent chalazion, ulcerative basal cell carcinoma, granulomatous blepharitis, and infected infundibular cysts [3, 10–12].

The diagnosis of cutaneous leishmaniasis is classically made with a tissue biopsy, which allows for the identification of the microorganisms through staining or culture [13]. In recent years, PCR has also emerged as a critical tool in diagnosis, offering the option for molecular analysis of specimens, which can be pivotal in guiding treatment strategies [14]. The effectiveness of these diagnostic methods was particularly significant in this case, as they led to the identification of the causative agent as *L. aethiopica.*

*L. aethiopica*, one of the most understudied species of Leishmania, is the predominant strain endemic to Ethiopia [15], where up to 65% of the population in affected areas is reported to have either an active or past infection of leishmaniasis [16]. The identification of this species was crucial, as *L. aethiopica* is known for its slower healing time and reduced sensitivity to conventional treatment regimens [17]. This knowledge underscores the importance of accurate diagnosis and species identification in the effective management of leishmaniasis cases.

In selecting treatment regimens for Leishmaniasis, it's crucial to consider the specific Leishmania species, patient toxicity risk, and local medication availability. Historically, pentavalent antimonials were the standard first-line treatment. However, due to rising resistance concerns, the WHO has updated its standard of care to recommend liposomal amphotericin B [18]. Recently, systemic oral miltefosine has emerged as an alternative treatment. It is often better tolerated than amphotericin B, which is associated with renal insufficiency and other serious side effects. Initially developed for cancer therapy, miltefosine has since received Food and Drug Administration (FDA) approval for treating three specific species of New World leishmaniasis and has been shown to be effective against L. aethiopica in several small clinical trials [19–21]. In this case, our patient's successful treatment involved a 28-day course of miltefosine.

Lastly, this case highlights the diagnostic value of a prompt skin biopsy, which would have undoubtedly led to a sooner diagnosis and treatment for the patient, as well as avoided unnecessary laboratory tests, diagnostic images, and healthcare expenses. Patient education and clear communication of risks and benefits of such procedures must be effectively achieved in order to achieve optimal outcome for all parties.

## Data Availability

Data sharing is not applicable as no datasets were generated for this study.

## Abbreviations

WHO: World Health Organization
CL: Cutaneous leishmaniasis
ACE: Angiotensin converting enzyme
IgG4: Immunoglobulin G4
RF: Rheumatoid factor
ANCA: Antineutrophil cytoplasmic antibodies
NIH: National Institutes of Health
FDA: Food and Drug Administration
PAS: Periodic acid-Schiff
GMS: Grocott’s methenamine silver

## Data availability

No datasets were generated or analysed during the current study.

## References

[1] Pahuja S, Puranik C, Jelliti B, Khairallah M, Sangwan VS (2013) Parasitic Infections of the External Eye. Ocul Immunol Inflamm 21:292–299. 10.3109/09273948.2013.770889 23617222 10.3109/09273948.2013.770889

[2] (2023) Leishmaniasis. World Health Organization

[3] Satici A, Gurler B, Aslan G, Ozturk I (2003) Ocular involvement in cutaneous leishmaniasis four cases with blepharoconjunctivitis. Eur J Epidemiol 19:263–266. 10.1023/B:EJEP.0000020346.15800.d3 10.1023/B:EJEP.0000020346.15800.d3 15117121

[4] Mignot G, Bhattacharya Y, Reddy A (2021) Ocular Leishmaniasis - A systematic review. Indian J Ophthalmol 69:1052. 10.4103/ijo.IJO_2232_20 33913831 10.4103/ijo.IJO_2232_20 PMC8186621

[5] Cohen JI (2013) Herpes Zoster. N Engl J Med 369:255–263. 10.1056/NEJMcp1302674 23863052 10.1056/NEJMcp1302674 PMC4789101

[6] Hauser A, Fogarasi S (2010) Periorbital and Orbital Cellulitis. Pediatr Rev 31:242–249. 10.1542/pir.31.6.242 20516236 10.1542/pir.31.6.242

[7] Curtin JM, Aronson NE (2021) Leishmaniasis in the United States: Emerging Issues in a Region of Low Endemicity. Microorganisms 9:578. 10.3390/microorganisms9030578 33799892 10.3390/microorganisms9030578 PMC7998217

[8] Jafari AK, Akhyani M, Valikhani M, Ghodsi ZS, Barikbin B, Toosi S (2006) Bilateral cutaneous leishmaniasis of upper eyelids: a case report. Dermatol Online J 12:20 16638388 10.5070/D39KQ7K2XN

[9] Mencía-Gutiérrez E, Gutiérrez-Díaz E, Rodríguez-Peralto JL, Monsalve-Córdova J (2005) Old World eyelid cutaneous leishmaniasis: a case report. Dermatol Online J 11:29 16409925 10.5070/D32PN8P44D

[10] Sadeghian G, Nilfroushzadeh MA, Moradi SH, Hanjani SH (2005) Ocular leishmaniasis: a case report. Dermatol Online J 11:19 16150227 10.5070/D34NH3H5NH

[11] Yaghoobi R, Maraghi S, Bagherani N, Rafiei A (2010) Cutaneous Leishmaniasis of the Lid: A Report of Nine Cases. Korean J Ophthalmol 24:40. 10.3341/kjo.2010.24.1.40 20157413 10.3341/kjo.2010.24.1.40 PMC2817823

[12] Veraldi S, Bottini S, Currò N, Gianotti R (2010) Leishmaniasis of the eyelid mimicking an infundibular cyst and review of the literature on ocular leishmaniasis. Int J Infect Dis 14:e230–e232. 10.1016/j.ijid.2009.07.024 19969498 10.1016/j.ijid.2009.07.024

[13] Aronson N, Herwaldt BL, Libman M, Pearson R, Lopez-Velez R, Weina P, Carvalho E, Ephros M, Jeronimo S, Magill A (2017) Diagnosis and Treatment of Leishmaniasis: Clinical Practice Guidelines by the Infectious Diseases Society of America (IDSA) and the American Society of Tropical Medicine and Hygiene (ASTMH). Am J Trop Med Hyg 96:24–45. 10.4269/ajtmh.16-84256 27927991 10.4269/ajtmh.16-84256 PMC5239701

[14] Aronson N, Herwaldt BL, Libman M, Pearson R, Lopez-Velez R, Weina P, Carvalho EM, Ephros M, Jeronimo S, Magill A (2016) Diagnosis and Treatment of Leishmaniasis: Clinical Practice Guidelines by the Infectious Diseases Society of America (IDSA) and the American Society of Tropical Medicine and Hygiene (ASTMH). Clin Infect Dis 63:e202–e264. 10.1093/cid/ciw670 27941151 10.1093/cid/ciw670

[15] Mengeot L, Yombi J-C, Baeck M (2022) Cutaneous leishmaniasis due to Leishmania aethiopica: A therapeutic challenge. JAAD Case Rep 20:72–75. 10.1016/j.jdcr.2021.12.028 35128017 10.1016/j.jdcr.2021.12.028 PMC8807949

[16] Bugssa G (2014) The Current Status of Cutaneous Leishmaniasis and the Pattern of Lesions in Ochollo Primary School Students, Ochollo. Southwestern Ethiopia. Sci J Clin Med 3:111. 10.11648/j.sjcm.20140306.13 10.11648/j.sjcm.20140306.13

[17] van Henten S, Adriaensen W, Fikre H, Akuffo H, Diro E, Hailu A, Van der Auwera G, van Griensven J (2018) Cutaneous Leishmaniasis Due to Leishmania aethiopica. EClinicalMedicine 6:69–81. 10.1016/j.eclinm.2018.12.009 31193672 10.1016/j.eclinm.2018.12.009 PMC6537575

[18] Alvar J, WHO, (2005) Report of a WHO informal consultation on liposomal amphotericin B in the treatment of visceral leishmaniasis. World Health Organization, Rome, Italy

[19] Ware JM, O’Connell EM, Brown T, Wetzler L, Talaat KR, Nutman TB, Nash TE (2021) Efficacy and Tolerability of Miltefosine in the Treatment of Cutaneous Leishmaniasis. Clin Infect Dis 73:e2457–e2562. 10.1093/cid/ciaa1238 33124666 10.1093/cid/ciaa1238 PMC8492130

[20] Alemu AY, Derseh L, Kaba M, Gadisa E, Alemu K (2023) Treatment outcomes of cutaneous leishmaniasis due to Leishmania aethiopica: A systematic review and meta-analysis. PLoS ONE 18:e0293529. 10.1371/journal.pone.0293529 37917604 10.1371/journal.pone.0293529 PMC10621858

[21] Van Griensven J, Gadisa E, Aseffa A, Hailu A, Beshah AM, Diro E (2016) Treatment of Cutaneous Leishmaniasis Caused by Leishmania aethiopica: A Systematic Review. PLoS Negl Trop Dis 10:e0004495. 10.1371/journal.pntd.0004495 26938448 10.1371/journal.pntd.0004495 PMC4777553

